# Current Evidence and Theories in Understanding the Relationship between Cognition and Depression in Childhood and Adolescence: A Narrative Review

**DOI:** 10.3390/diagnostics12102525

**Published:** 2022-10-18

**Authors:** Stefano Barlati, Jacopo Lisoni, Gabriele Nibbio, Giulia Baldacci, Andrea Cicale, Laura Chiara Ferrarin, Mauro Italia, Andrea Zucchetti, Giacomo Deste, Antonio Vita

**Affiliations:** 1 Department of Clinical and Experimental Sciences, University of Brescia, 25123 Brescia, Italy; 2 Department of Mental Health and Addiction Services, ASST Spedali Civili of Brescia, 25123 Brescia, Italy

**Keywords:** adolescent, at-risk subjects, cognitive functions, cognitive vulnerability, cold cognition, hot cognition, depression, early detection, staging model

## Abstract

The present narrative review has covered the current evidence regarding the role of cognitive impairments during the early phase of major depressive disorder (MDD), attempting to describe the cognitive features in childhood, adolescence and in at-risk individuals. These issues were analyzed considering the *trait*, *scar* and *state* hypotheses of MDD by examining the *cold* and *hot* dimensions, the latter explained in relation to the current psychological theoretical models of MDD. This search was performed on several electronic databases up to August 2022. Although the present review is the first to have analyzed both *cold* and *hot* cognitive impairments considering the *trait*, *scar* and *state* hypotheses, we found that current evidence did not allow to exclusively confirm the validity of one specific hypothesis since several equivocal and discordant results have been proposed in childhood and adolescence samples. Further studies are needed to better characterize possible cognitive dysfunctions assessing more systematically the impairments of *cold*, *hot* and *social cognition* domains and their possible interaction in a developmental perspective. An increased knowledge on these topics will improve the definition of clinical endophenotypes of enhanced risk to progression to MDD and, to hypothesize preventive and therapeutic strategies to reduce negative influences on psychosocial functioning and well-being.

## 1. Introduction

A critical stage for the manifestations of severe mental illness (SMI) is adolescence, as it represents a period of remarkable physical and behavioral changes during which neurodevelopment and neural maturation occur [1]. Among these modifications, the increased myelination and connectivity of white matter boundaries, and the corresponding decreases of grey matter regions, result in increased neuronal efficiency and specialization [2]. During this period, subtle or subthreshold symptoms could occur and, if not adequately recognized and treated, may produce persistent and disabling manifestations of mental disorders including depressive symptoms [3,4,5,6] or other psychiatric comorbidity [6], such as self-harming behaviors and pathological drinking [7,8,9].

Among these heterogeneous clinical features, cognitive disturbances are central elements not only during the established course of a specific SMI such as schizophrenia [10], depression [11] and bipolar disorder (BD) [12], anxiety disorders, obsessive-compulsive disorder [13] or eating disorders [14], but also during the early phase or even during the premorbid stage of impelling mental disorders [15,16,17,18]. This undoubtedly produces negative repercussions on psychosocial and functional outcomes, disability levels and quality of life of young affected people [6,19,20,21]. It is therefore not surprising that, in recent decades, neuropsychology has been established as an essential discipline in psychiatric settings to assess youth patients’ psychopathological needs and to orient clinical formulation and therapeutic interventions [22].

Generally speaking, these cognitive impairments can be described as belonging to three different domains. While the *cold* cognitive domain includes cognitive abilities that are independent from emotional involvement (i.e., attention, memory and learning abilities, cognitive flexibility, planning and working memory), the *hot* cognitive domain includes cognitive abilities influenced by emotional stimuli (i.e., reward learning, decision-making and risk-taking processing). Finally, the *social cognition* domain includes cognitive abilities related to interpersonal contacts and to the perception of oneself and others in the social environment, such as emotion perception, theory of mind (ToM), and attributional style [22,23,24,25]. However, while in disorders such as schizophrenia, a solid consensus has been reached regarding neuropsychological impairment during all the phases of the disorder [23,24,25,26]. On the other hand, scientific evidence on cognitive disturbances in major depressive disorder (MDD) has only recently been re-evaluated, especially when considering young populations.

In further detail, MDD is one of the most prominent mental health disorders affecting adolescents, with a 1-year prevalence of 4% to 5% worldwide [27]. Moreover, while depressive symptoms seem to be at low level during late childhood/early adolescence up to age 11, then increasing at around age 13 and worsening between ages 15 and 18 [28,29], it also appears that rates of depressive symptoms after age 18 tend to level off, remaining relatively stable throughout most of adulthood [28,30]. However, important individual differences in depressive symptom trajectories have been reported, with evidence of both continuity and change in depressive symptoms during adolescence [31,32].

Among clinical manifestations of MDD, cognitive deficits represent a main feature of the disorder although they have been historically considered a consequence or a residual phase symptom that followed an acute episode [33,34]. More precisely, current conceptualizations suggested that disturbed neuropsychological functioning does not simply represent an epiphenomenon of depression (that is, it is not a merely secondary symptom of an acute depressive episode), while it should be considered, more properly, a primary core feature being present throughout all the phases of the disorder [33,34]. Indeed, cognitive impairments are present during the acute depressive phase, affecting executive functions (EF), attention, memory and psychomotor speed [35,36]. Congruently, meta-analytic results confirmed that first-episode MDD patients showed psychomotor speed, attention, visual learning and memory, and EF impairments [16]. Moreover, it was also observed that the impairment reported in the acute phase of the disorder could be long lasting despite symptom recovery [35]; thus, the meta-analytic evidence strongly confirmed persistent poorer cognitive functioning also during the euthymic phases of the disorder, involving processing speed, attention, working memory and EF impairments if compared to healthy controls (HC), with MDD late-onset cases resulting in more pronounced impairments [37]. Summing up, it has been found that cognitive symptoms in MDD are consistent, replicable, clinically significant, albeit nonspecific and of small to medium in effect size, and could include either deficits of one or more cognitive domains. Particularly, if impairments of *cold* cognitive domain comprise several disturbed cognitive processes on neutral affective cues (i.e., attentional, memory, EF dysfunctions), impairments in the *hot* cognitive domain are related to negative inferential/attribution styles, aberrant attentional allocation processes toward negative-valence stimuli, abnormal interpretation of social stimuli and ruminations rather than a temperamental individual predisposition to MDD [33,34,38]. Finally, integrating some aspects of both *cold* cognition and *hot* cognition, disturbances of *social cognition* domain represent another important cognitive dimension in MDD, encompassing the perception, identification, and interpretation of social stimuli, including facial expressions, verbal/non-verbal cues as well as the mental states of others (ToM) [34,38].

Nevertheless, if the course of cognitive disturbances after the first depressive episode has been widely studied across adult cohorts, less attention has been paid to cognitive impairments when depression occurs during childhood and adolescence or even in a premorbid phase, although this population is at high risk of recurrence [39] with severe long-term psychosocial impairment [40]. While numerous explanatory theories have considered the impact of biological factors (including altered neurotransmission, genetics factor, endocrine alterations), psychological factors (including cognitive vulnerability, ruminations, negative interpersonal relations, erroneous coping styles, emotional reactivity, negative affectivity) and socio-cultural factors (including stress exposure) to explain the increased prevalence of depression in childhood and adolescence [27,41], the causes and consequences of cognitive impairments in MDD, especially in youths, are still questioned. Indeed, it is complex to assess the contribution of any single risk factor and to identify a specific developmental frame time during which cognitive impairments could manifest [27] since distal and proximal familial, genetic, and psychosocial factors act mediating their effects through temperament and personality attributes (negative emotionality, decreased positive emotionality and attentional control, behavioral inhibition, and neuroticism), and cognition [27,29,42]. In this context, a more extensive identification of risk factors, illness progression and barriers to recovery is essential to provide effective early interventions among young MDD patients or in the at-risk stage.

### Aims

Among the cognitive disturbances in MDD, impairments of the *cold* cognitive domain represent an area of clinical interest for many important reasons [38]. First of all, cognitive functioning could be considered as a predictor of treatment outcomes [43,44,45]. More specifically, cognitive dysfunctions have been associated, both in adult and youth depressed patients, to poorer response to treatments [46,47,48,49,50], to poorer social/vocational functioning and greater disability [51,52,53,54] and to an increased risk of suicide [55,56,57,58], impeding functional recovery from MDD [59]. Furthermore, as *cold* cognitive impairments could represent a suitable biomarker of vulnerability [60], a longitudinal predictor of socio-occupational functioning among young psychiatric outpatients [51] and a predictor of recurrence of new depressive episodes [61], impairments of *cold* cognitive domain have been deemed as an essential therapeutic target to ensure functional recovery following a depressive episode [59]. Therefore, taking into account these premises, attempting to cover the antecedent cognitive characteristics in childhood and adolescence that could increase the risk of depression, the present review is aimed at investigating whether, in youth population during the early stages of MDD or in the pre-morbid stages, the identification of impairments in *cold* cognitive domains is a feasible area of research and whether this translates to utility in clinical practice. Moreover, we are also interested in considering possible impairments in the domain of *hot* and *social cognition.* We considered the topic of identifying potential cognitive deficits in young people as crucial as it has several practice implications regarding the possibility to provide further preventive or therapeutic strategies aimed at reducing the impact of these impairments on psychosocial functioning and well-being, by modifying the trajectory of MDD, or postponing or avoiding its onset.

**Figure 1:**
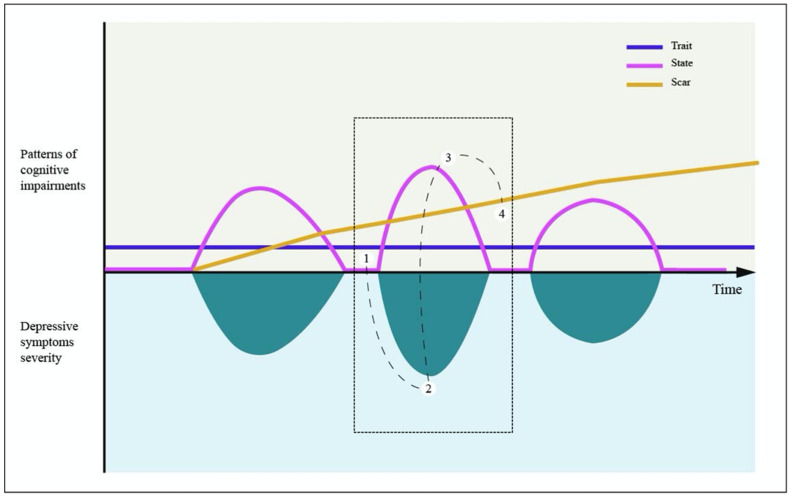
A possible model of cognitive impairments in major depressive disorder (MDD) according to *trait*, *scar* and *state* hypotheses. The central rectangle (dots lines) describes a model of possible interaction between *cold* and *hot* cognitive domains during an acute depressive episode: (1) triggered by stressful and/or negative events, *cold* cognitive impairments (mainly of the executive functioning) could activate cognitive biases and maladaptive schemata; (2) when an acute depressive episode occurs, depressive symptoms exacerbate cognitive impairments by turning and incorporating *cold* cognitive deficits into the expression and maintenance of *hot* cognitive biases; (3) the depletion of cognitive resources allocated to everyday functioning are further depleted by cognitive biases and maladaptive emotion regulation (i.e., ruminations) prolonging the depressive mood state further contributing to broader cognitive deficits across several *cold* domains; (4) during the remission phase of a depressive episode, persistent *cold* cognitive impairments (mainly in executive functioning) act as trait or scar-like impairments leading to a recurrence of the depressive cycle, especially when stressing mediators (personal internal/external factors) restart *hot* cognitive disturbances (i.e., maladaptive schemata and ruminations). The model is adapted from Allot et al., 2016 [60] and Ahern et al., 2019 [38].

To our aims, as it is necessary to define a theoretical framework to understand the possible nature and source of cognitive impairments in young populations, the *trait*, *scar* and *state* hypotheses (Figure 1) is a useful framework aimed at responding to whether neurocognitive impairments are pre-existing trait/vulnerability markers, state-related impairments or scar impairments [60,62]. Since the aim of the present review is to inform the reader about the feasibility to identify possible cognitive impairments during the premorbid phases or early stages of MDD in childhood/adolescence, particular attention will be given to current evidence regarding *cold* and *hot* cognitive functioning by considering the potential trait-like nature of these impairments.

## 2. Materials and Methods

### Search Strategy and Study Selection

We explored the research topic by searching relevant publications throughout several electronic databases (Google Scholar, PubMed, Scopus and Web of Science), from database inception to 16 August 2022. Search strategy was based on the combination of several keywords: “depression” AND “adolescence” OR “childhood” OR “at risk” OR “pre-morbid” OR “familiar risk” AND “cognitive impairment” OR “cold cognition” OR “hot cognition” OR “social cognition” OR “trait hypothesis” OR “state hypothesis” OR “scar hypothesis” OR “cognitive biases” OR “rumination” OR “negative inferential styles” OR “intelligence quotient” OE “memory” OR “attention” OR “executive functions”.

To be included in the present review, eligible papers had to meet the following criteria: (1) to be published in peer-reviewed journals; (2) to be available in the English language; (3) no restrictions on study design were considered (i.e., longitudinal, prospective, cross-sectional studies, and systematic/narrative reviews, and meta-analysis). Moreover, to be included in the review, study samples should have involved (4) children, adolescents or young adults (aged <25 years old) having (5) (with/without) familiar or environmental risk factors for MDD, (6) (current/past) history of MDD (according to *previous and current* versions of the *Diagnostic and Statistical Manual of Mental Disorders and the International Classification of Diseases)*, including being euthymic or in a pre-morbid phase of the disorder, and (6) (with/without) other psychiatric comorbidities. Exclusion criteria were the following: (1) papers not published in English; (2) papers not published in peer-reviewed journals; (3) study samples comprising individuals with schizophrenia or bipolar spectrum disorder as a primary diagnosis (according to *previous and current* versions of the *Diagnostic and Statistical Manual of Mental Disorders and the International Classification of Diseases)*. No restriction regarding year of publication was established.

Emerging reports were independently assessed by at least two reviewers (among J.L., G.N. and S.B.). To obtain additional information, the same authors manually inspected reference lists of included papers and review articles emerging from the search. The identified abstracts were inspected, then the full texts of eligible studies were again assessed for accuracy by at least two reviewers (among G.B., A.Z., A.C., L.F., M.I.), who also extracted relevant data from selected papers.

## 3. Results

### 3.1. The Trait Hypothesis

The *trait* hypothesis suggests that neurocognitive impairments and cognitive vulnerability are presumably present prior to depressive symptoms onset, by means as contributing to increase the risk of developing depression. Moreover, the *trait* hypothesis considers this vulnerability as a predisposition that persists during symptomatic remission, thus being independent of a clinical state, and further representing a risk factor for future relapse [60,62]. Hence, trait impairments may occur through biological (heritable or not) or environmental mechanisms [60]. While evidence for the trait neurocognitive impairment hypothesis should come from studies investigating neurocognitive functioning of individuals in the early phases of the disorder before development of full-threshold first-episode MDD, current data are generally sparse and inconclusive, mostly because high-risk studies were generally performed in adults rather than in already symptomatic youths or included individuals with other psychiatric comorbidities.

A first consideration comes from studies that considered the unaffected relatives of individuals with MDD, with the idea that the presence or the absence of cognitive impairment in this at-risk population would suggest that impaired cognition may represent a precursor or a consequence, respectively, of MDD. A recent meta-analysis by Mackenzie et al. [63] included 3.246 relatives of people with MDD (mean age 15.38 years) and 5.222 controls (mean age 14.70 years), showing that relatives of people with MDD performed worse than controls across all measures of cognition. Specifically, first-degree relatives of individuals with MDD performed significantly worse on global IQ measures, verbal intelligence, perceptual intelligence, memory, academic performance and language tests, confirming the evidence of a slightly but significantly impaired cognition in this population compared people without a family history of SMI. Indeed, it also reported small differences between first-degree relatives of people with MDD and controls in nearly all cognitive domains, suggesting that familial liability to depression could be associated with a broad impairment in cognition rather than a distinct cognitive profile; these results likely reflect the contribution of several genetic, social and environmental factors related to the risk of MDD [63]. Another meta-analytic study [61], including results on studies on 121.749 children, adolescents and adult individuals, showed that higher cognitive functioning was associated with decreased levels of subsequent depression, although this association was driven by concurrent depressive symptoms at the time of cognitive assessment. The authors argued that cognitive deficits predicting MDD could be described, at best, as being a deleterious factor of subclinical depression symptoms on cognitive performance rather than a pure premorbid risk factor for MDD development [61].

Supporting the *trait* hypothesis, Belleau et al. [64] studied a group of youth (mean age 16 years) having or not one parent with either BD or MDD on a cognitive battery assessing alerting, orienting and executive attention; it was found that only those individuals at familiar risk for mood disorder showed significantly slower reaction times on executive attention, but not on alerting or orienting. Another longitudinal 9-years study by Vinberg et al. [65] on 234 healthy monozygotic and dizygotic twins with and without a co-twin history of affective disorder found that impairments of executive function at Trail Making test A-B and low attentional and language tasks scores at the Cambridge Cognitive Examination-Revised could predict the subsequent onset of future depression development. Similarly, Simons et al. [66] evaluated cross-sectionally and prospectively the association between depressive symptoms and neuropsychological functioning of episodic memory and information processing in a cohort of 569 female twins and 43 of their sisters, showing that future depressive symptoms were strongly associated to low neuropsychological functioning. Particularly, it was found that, if poor information processing could be the result of an acute depressed state, poor episodic memory functioning could represent a predictor of future depressive symptoms. Anyway, it must be considered that while studies of twins could provide a powerful endorsement to the *trait* hypothesis, a main limitation is that these studies have been generally carried out in an adult population, thus making it difficult to assess the premorbid cognitive status in a proper at-risk population of childhood or adolescent individuals [60].

More recently, considering the visuospatial processing as a core element of EF, Singh et al. [67] were interested in studying if early deficits were present before the onset of a mood disorder or were related to risk for MDD rather than BD. They studied 111 children, aged 8–17, of parents with BD or with MDD, and demographically healthy control youths without any family history of psychopathology with the Delis–Kaplan Executive Functioning System Trail Making Test. They found that youths at familial risk for BD or MDD had a significantly lower performance on visual scanning, number-sequencing, letter-sequencing, and number-letter switching than HC youths, suggesting that these impairments could precede the onset of a mood [67]. Consistent with the notion that MDD is associated with a broad impairment of multiple aspects of executive functioning [68], Han et al. [69] longitudinally studied 220 adolescents, aged 11–16, and their parents, assessing executive functioning by the Wisconsin Card Sorting Test (WCST) over a 2-year period, showing that youths with higher levels of global executive functioning impairments reported, prospectively, significantly more frequent concurrent depressive symptoms. The authors also noted that having more global executive functioning deficits could predict youth to exhibit more anxious symptoms two years later [69]. Congruently, Halse et al. [70] showed that reduced executive functioning could predict symptoms of MDD but also of other psychiatric disorders, including anxiety, ADHD, oppositional defiant and conduct disorder in a sample of 874 children and adolescents, aged 6–14, followed over 2 years.

Moreover, Hawkey et al. [71] studied prospectively 247 children, aged 3–6, confirming that premorbid executive functioning during childhood could predicted the onset or worsening of both ADHD and depression, probably acting as an early common liability risk factor for the development of ADHD and depression, which are often comorbid conditions [72]. In this context, since ADHD is a primary cognitive disorder, it is not surprising that ADHD could represent as a candidate to investigate if cognitive symptoms may act as antecedent risk factors to further develop a depressive disorder [33]. Indeed, in a prospective study by Biederman et al. [73], 140 adolescent and young adult female individuals with ADHD and 122 without ADHD were assessed for cognitive (i.e., IQ at the Wechsler Intelligence Scale for Children—Third Edition, WISC-III), social and educational functioning over a period of 5 years to evaluate the possible associations between ADHD and MDD, finding that ADHD was associated with impaired cognitive and educational outcomes and that the presence of ADHD provided a 2.5 times higher risk to develop future MDD episodes. Thus, the authors suggested that ADHD cognitive symptoms could represent a risk factor for the development of future depressive symptoms [73].

Similar results have been obtained considering studies including patients with a full-blown affective disorder. Indeed, Peters et al. [74] assessed longitudinally a group of 62 youths in remission from MDD (aged between 18–23 years) and healthy controls with a series of neuropsychological tests on several neurocognitive domains. They found that youths with MDD in remission performed comparably to healthy controls on verbal fluency, processing speed and set-shifting, but not on cognitive control measures. The authors therefore suggested that deficient cognitive control might represent a trait vulnerability or an early course scar of MDD that may prove a viable target for prevention and early remediation [74]. Although quite beyond the scope of the present review, Sarapas et al. [75] considered data from a 26-year longitudinal study on 42 unipolar and 47 bipolar adult participants, showing that individuals with higher past average depression severity showed greater decrements in attention/psychomotor processing speed and, among unipolar individuals, in cognitive flexibility. Moreover, the authors found that cognitive performance on all examined domains was stable over the course of six years and was independent of affective symptom fluctuation, with bipolar individuals performing worse than unipolar ones on attention/psychomotor processing speed. Given the stable relationship between mood disorder severity and cognitive deficits, Sarapas et al. concluded that these findings were consistent with the *trait* hypothesis between overall mood disorder severity and neuropsychological impairment, suggesting, on the contrary, that state changes of depressive symptoms appeared to have minimal influence on cognitive performance [75].

In opposition to the *trait* hypothesis, Klimes-Dougan [76] found that children of mothers with BD showed deficits in executive functioning and selective deficits in spatial memory and attention, in comparison with children of healthy mothers, while memory deficits were not detected in high-risk children of MDD mothers, suggesting that these deficits may be a state marker of MDD. Similarly, Maalouf et al. [77] showed that impaired EF and impulsivity were not a trait marker in adolescent MDD, resembling more a state-specific marker since they were present during an acute MDD episode and did not persist during the remission phase. Furthermore, the authors highlighted that these deficits were associated with depression severity in adolescents with a history of MDD. More recently, Schaefer et al. [78] disconfirmed the *trait* hypothesis by demonstrating that childhood cognitive functioning and IQ did not predict future risk of MDD and also failed to confirm the *scar* hypothesis by demonstrating that low cognitive functioning was not an enduring consequence of an MDD episode, since participants with a past history of MDD did not show evidence of greater cognitive decline, unless MDD was accompanied by other comorbid psychiatric conditions. Thus, it was hypothesized that low cognitive functioning was almost related to other psychiatric comorbidities [78].

Partially supporting the *trait* hypothesis, Koenen et al. [79] found that for each standard deviation increase in childhood IQ, there was a 23% reduction in the odds of having an adult MDD diagnosis, while lower IQ was associated with greater risk of persistent depression in adulthood. Moreover, Glaser et al. [80] investigated the association between IQ assessed at age 8 years with depressive symptoms at 11, 13, 14, and 17 years, showing a positive association between childhood IQ and depressive symptoms in adolescence, although the direction of the relationship varied according to age and pubertal stage, with lower IQ to be associated with higher depressive symptoms at age 11, and higher IQ to be associated with higher depressive symptoms at different time points.

Summing up, if lower IQ seems to have some association with an increased risk for MDD, other evidence regarding specific neurocognitive domains as possible risk factors for MDD in youths are limited and inconclusive. Thus, other authors provided a more complex explanation, disentangling the role of other possible mediators. By assessing annually 523 euthymic adolescents, Giollabhui et al. [81] evaluated if impaired attentional functioning could be a risk factor for depression, a consequence of it, or whether both depression and impaired cognition were caused by a third underlying process (e.g., stress). Firstly, the authors found that switching attention, and minimally selective attention, declined prior to the depression onset, subsequently recovering in the years following the depressive onset. Moreover, they found that impaired switching attention prospectively predicted higher depressive symptoms and that higher depressive symptoms predicted worse selective and switching attention. Thus, Giollabhui et al. suggested a complex, reciprocal interaction between depressive symptoms and attentional functioning, driven by the exposure to childhood stress as a factor that may explain the relationship between depression and cognitive functioning; in this context, childhood stress could predict more severe depressive symptoms via impaired switching attention and vice versa [81].

On the other hand, taking into account the distinction between *cold* and *hot* cognition, it was highlighted that if neurocognitive functioning could be characterized by an absence of *cold* cognitive deficits prior to the first depressive episode, a trait-like vulnerability could be considered in light of the *hot* cognitive tendency to attribute stressful life events to global, stable and internal causes. In the presence of perceived stress, it seems that negative cognitive styles could trigger a depressive episode, presenting as a dysregulation of *hot* cognition where the individual’s focus on analyzing the negative aspects of the environment and the self leads to fewer resources being available for other cognitive processes [82].

### 3.2. The Scar Hypothesis

On the other hand, it has been suggested that depression is responsible for a neurotoxic action at a developmental level, leading to irreversible impairments of cognitive functioning, with progressive worsening explained as a consequence of the chronicity and severity of affective episodes [60,62] and linked to dysregulated neurobiological processes (including of the HPA-axis, inflammation, oxidative repair, apoptosis) acting as central mechanisms interfering with the physiological progression of neurogenesis [60,83]. However, differently to adult individuals, cognitive impairment in the depressed youth population may not necessarily manifest with a clear decline over time, and it could rather present through an attenuation of neurocognitive performance, becoming evident when the individual’s functioning is compared to that of healthy peers [60].

Firstly, as they failed to find deficits of EF facets and attention in children and adolescents with MDD, Vilgis et al. [84] supported the *scar* hypothesis by suggesting that such impairments require time to manifest as they worsen and as the severity and chronicity of affective episodes increase. Recently, Vijayakumar et al. [85] conducted a prospective study assessing the relationship between cognitive control impairments and the onset of MDD during early and late adolescence in a cohort of 165 adolescents without a current or past history of MDD. In this study, participants completed a cognitive control task at baseline and 4 years later. The authors found that cognitive control changes differed depending on the timing of MDD onset; precisely, cognitive control improved in accordance with normal development in adolescents who either did not develop MDD or who developed MDD in late adolescence. In contrast, an arrest of cognitive control development was found in participants that experienced MDD during early adolescence. Thus, it was argued that the normal development of cognitive control in the late-onset MDD group could support the *scar* hypothesis [85]. Another longitudinal study by Beaujean et al. [86] assessed cognition prior to the onset of MDD in a cohort of 14.322 adolescents to investigate the relationship between depression and cognitive ability at baseline and after 8 years; partially supporting the *scar* hypothesis, it was found that depressive symptoms in adolescence were related to cognitive ability in early adulthood, although cognitive ability in adolescence was not related to adult depression levels. Thus, the authors observed that the relationship between cognitive ability and depression was present only during the early life stages disappearing further in adulthood. Moreover, it was suggested that lower cognitive ability in adolescence due to depressive symptoms could be restored by intervening in early stages of depression [86].

Considering self-esteem as a predictor of depressive episodes development (according to the *trait* hypothesis) rather than a consequence of persistent depressive symptoms (according to the *scar* hypothesis), Steiger et al. [87] studied longitudinally a cohort of 1.359 individuals, from adolescence to middle adulthood and across two generations, showing that adolescent self-esteem and depressive symptoms were prospectively related to adult self-esteem and depressive symptoms three decades later, thus suggesting that both the *trait* and *scar* models are valid over decades with stronger evidence for the *trait* hypothesis.

In opposition, Seinberger and Barch [88] longitudinally assessed a sample of 11.878 children, aged 9–11, to analyze the possible role of motivation as a mediating factor of the relationship between depression and cognition with the aim to further clarify the state, trait and scar nature of cognitive deficits in depression in a late childhood sample. The authors found no evidence of concurrent state or longitudinal trait or scar relationship between depression and cognition. Indeed, no support was found of depression as being related to concurrent or subsequent cognitive worsening. Moreover, when accounting for comorbid anxiety, no evidence of a prospective relationship between cognitive functioning and the onset of depression was found. Finally, no evidence was found regarding disruptions in motivation as a possible mediator of the relationship between depression and cognition. Specifically, as no significant relationship between composites score of cognition over time and baseline depression was found, the authors concluded by rejecting the *scar* hypothesis of cognitive dysfunction in MDD [88]. Similarly, Stange et al. [89] argued that depression did not elicit scarring effects on cognitive ability: assessing a never-depressed sample of 285 adolescents over 4 years with self-report and behavioral measures of rumination and attentional shifting, the authors found that cognitive inflexibility (i.e., higher levels of rumination and poorer attentional shifting abilities) could predict a shorter time to first onset of major depression, thus representing an important risk factor for the onset of MDD during adolescence. Moreover, indirectly supporting the *trait* hypothesis, it was found that rumination and attentional shifting were not correlated, acting as independent predictors of depression onset.

### 3.3. The State Hypothesis

The *state* hypothesis argued that cognitive impairments are caused by the depressive symptom state. In other words, temporary cognitive dysfunctions represent a state feature of acute depression that will increase or decrease with exacerbation or resolution of depressive symptomatology, normalizing parallelly with affective symptom improvement [60,62]. Moreover, cognitive impairments are expected to be more severe with greater symptom severity and may occur over and above existing trait or scar impairments [60]. Indeed, if previous studies have clearly demonstrated the cognitive impairments of adolescent and young adult individuals with current MDD [16,90], a limitation of these studies comes from the inability to distinguish state-like from trait-like neurocognitive deficits, completely excluding the scar-like effects. Moreover, an actual limitation is that most of these studies assessed MDD individuals ranging between young and middle adulthood, while only few studies considered children/adolescent population. Furthermore, it is questionable to verify the *state* hypothesis by considering an at-risk sample prior to the depression onset. Thus, since the purpose of this review is to evaluate the possible role of cognitive deficits as antecedents to depression in at-risk individuals, we consider that investigating the *state* hypothesis perspective is beyond our scope and of limited interest.

Some relevant findings regarding this hypothesis, however, have to be discussed. Micco et al. [91] assessed 147 at-risk offspring, aged 6–17, of parents with MDD and/or panic disorder (PD) and of controls with neither disorder, with the aim at understanding whether EF impairments could predate the onset of disorder, or if they reflected acute symptoms. They found that, although parental MDD and PD were not associated with neuropsychological impairments, the presence of current MDD in offspring was associated with poorer performance on several executive functioning and processing speed measures, suggesting that EF deficits could not serve as a trait marker for developing anxiety or depression, appearing as symptoms of the current disorder. Maalouf et al. [77] conducted a cross-sectional study to examine the state versus trait markers of MDD by comparing the cognitive performance on EF, sustained attention and short-term memory in 20 adolescents with MDD in acute episode, 20 depressed adolescents in remission and 17 healthy controls; the authors found significant EF impairments in the acutely depressed sample compared to those in remission and healthy controls, with higher impulsivity scores associated with more severe depression and earlier age of onset of depression. Subsequently, Maalouf et al. [92] assessed if bias to negative emotions in an inhibitory control paradigm would be considered a state or trait marker in MDD adolescents; using an affective go/no go task with 40 adolescents with acute MDD, 20 depressed adolescents in remission and 17 healthy controls, it was found that bias to negative emotional stimuli was present in the acute stage of MDD and absent in remission, advising that it was a state-specific marker of depressed adolescents.

### 3.4. Cognitive Vulnerability as an Index of Hot Cognitive Dimension in Depression: The Role of Cognitive Biases

In recent years, a growing interest regarding cognitive vulnerability theories, within a developmental framework has led to rapid advances in prospective studies testing cognitive theories of vulnerability to depression in child and adolescent samples. Thus, it is not surprising to note that individual cognitive biases were significantly associated with depression severity, particularly when a combination of cognitive biases, that include interpretation bias and negative self-evaluation, is present [93]. Generally speaking, based on diathesis–stress models that considered depression as the result of the interaction between an individual’s cognitive vulnerability and triggering environmental situations, these cognitive theories have defined the concept of cognitive vulnerability as an individual’s internal and stable feature that could predispose to the development of further depression. Thus, when a negative event occurred during the life of a person that possesses a cognitive vulnerability, a pattern of negatively biased and self-referent information processing could be triggered, initiating a downward spiral into depression [42]. In this context, cognitive vulnerability, negative events and depression are considered along a continuum of severity, so that the higher the level of cognitive vulnerability an individual presents, the less stressful the negative event that could elicit the onset of depressive symptoms [42]. Furthermore, in a developmental perspective, it has been suggested that cognitive vulnerability factors could moderate the relationship between stress and depression, especially during the transition from childhood to adolescence, that is, precisely when individuals are faced with greater levels of possible negative or stressful experiences and when greater cognitive skills are required to attempt to reach socio-educational goals [42]. Thus, several findings previously supported a trait-like nature of these depressogenic cognitive vulnerability factors [94,95] since they have found to be stabilized, to some extent, during early adolescence [96]. In line with current evidence [38], we present these cognitive disturbances considering their involvements in the *hot* cognitive domain.

Among cognitive vulnerability factors, depressogenic inferential styles are described by the hopelessness theory [97]—individuals may attribute negative life events to stable (enduring) and general causes, catastrophizing about the consequences of a current negative event, and inferring that the occurrence of a negative event in their lives means that they are deficient or unworthy. Individuals who exhibit such negative inferential styles should be more likely to make negative inferences about the causes, consequences, and self-implications of any negative event they encounter, thereby incrementing the likelihood of becoming hopeless, the proximal symptom of an emerging depression [98]. While Hankin [99] found that a negative inferential style interacted with negative events to predict symptoms of general and anhedonic depression in a sample of 350 adolescents, more recently Giollabhui et al. [100] demonstrated the validity of the hopelessness model of depression in order to predict first onset of a MDD episode. In a community sample of adolescents, aged 12–13, they found that hopelessness mediated the relationship between negative inferential style and depression (both depressive symptoms and first onset of a MDD episode) at higher levels of many forms of negative life events (NLEs), confirming that NLEs were a necessary precondition to activate a negative inferential style, thereby leading to hopelessness.

Another cognitive vulnerability factor is represented by dysfunctional attitudes [101]. According to Beck’s model, negative self-schemata, organized around themes of failure, inadequacy, loss and worthlessness, could serve as a vulnerability factor for the depression onset and exacerbation. Such negative self-schemata were often represented by dysfunctional attitudes according to which the individual is prone to believe that happiness and worth depend on being perfect or on others’ approval [42]. Although several prospective studies examined the cognitive vulnerability hypothesis of Beck’s theory in adolescent samples, only limited support is given to this construct [102]. Lewinsohn et al. [103] assessed longitudinally more than 1.500 adolescents concluding that, supporting Beck’s theory and refusing the hopelessness theory, findings were suggestive of a threshold view of vulnerability to depression—for those who experienced NLEs, depressive onset was related to dysfunctional attitudes but only when dysfunctional attitudes exceeded a certain level.

A third cognitive vulnerability factor is described by the Response Styles Theory [104] according to which individual responses (i.e., rumination and distraction) to symptoms of depression determines both the severity and duration of depressive symptoms: indeed, individuals who tend to ruminate in response to depressed mood are at greater risk for experiencing prolonged and severe depressive episodes compared to individuals who tend to distract themselves [60,62]. Several prospective studies have examined this theory in youth populations, generally supporting that rumination is associated with greater severity of depressive symptoms over time [60,105]. Particularly, Kuyken et al. [106] assessed longitudinally a sample of 326 adolescents, aged 14–18, as either at normal or increased risk for depression, showing that at-risk adolescents were characterized by more severe rumination levels than adolescents who were not at risk. Similarly, Hankin [107] found that baseline rumination prospectively predicted the fluctuations of depression symptoms and of general internalizing problems in a sample of 350 adolescents. Moreover, Driscoll et al. [108] found that a ruminative response style was associated with an increased risk of depressive symptoms for children who encountered increased levels of stress and, especially, for female individuals that reported higher rumination scores than boys, reflecting greater use of rumination relative to distraction. More recently, Cohen et al. [109] longitudinally studied 473 early and middle adolescent students that completed self-reports of cognitive vulnerability and depressive symptom indexes at baseline and every 6 months for 3 years. The authors found that rumination was the unique predictor for the first depressive episode, while for recurrent major depression rumination in early adolescence and attributional style in middle adolescence were identified as incremental predictors beyond baseline depressive symptoms.

Furthermore, an attempt to disentangle the relationship between *hot* cognitive vulnerabilities and *cold* cognition impairment comes from to the resource allocation theory, according to which negative thoughts of depression and rumination could compromise further cognitive abilities that would otherwise be directed towards task-relevant processes, specifically affecting executive functioning (i.e., cognitive control and attentional abilities) [110]. In this scenario, Connolloy et al. [111] evaluated longitudinally 200 adolescents, aged 12–13, to assess if higher levels of rumination and depressive symptoms at baseline could predict, at a 15-month follow-up, impairments on executive functioning on neutral-cue attention and memory tasks. Partially supporting the resource allocation hypothesis, the authors highlight the potential effects of ruminations on EF during early adolescence, showing that higher levels of baseline rumination (but not of depressive symptoms) prospectively predicted decreases in selective attention and attentional switching at follow-up, while lower levels of EF at baseline did not predict a further increase of rumination and depressive levels. More recently, Wagner et al. [112] assessed 486 early adolescents and their mothers/primary caretakers to examine if trait rumination significantly predicted attentional set shifting and sustained attention functioning and if impaired executive functioning was associated with unipolar MDD diagnosis or current depressive symptoms. Contrary to what they expected, they found that current depressive symptoms moderated the rumination-sustained attention association, such that higher rumination levels predicted better sustained attention in those with lower depressive symptoms and worse sustained attention did so in those with higher depressive symptoms. Moreover, the Authors found that higher depressive symptoms marginally predicted poorer sustained attention but did not affect any EF measure, thus rejecting the hypothesis that current depressive symptoms act as a mediating factor in executive functioning impairments.

Summing up, several previous studies demonstrated the possible associations between cognitive vulnerability factors (i.e., negative inferential styles, dysfunctional attitudes and ruminative response styles) and depression [109,113,114] leading to negative impacts on MDD course with an increased risk of higher severity and recurrence [109,115,116].

## 4. Discussion

The present review covered current evidence regarding the role of cognitive impairments during the early phase of MDD, attempting to describe the cognitive features in childhood and adolescence that could increase the risk to later develop depressive episodes. We analyzed these issues considering *trait*, *scar* and *state* hypotheses (Figure 1), as these models could help to disentangle whether cognitive impairments are pre-existing trait/vulnerability markers, scar-like or state-like impairments [60]. Indeed, these hypotheses are relevant to understand cognitive profiles of MDD in youths at risk for depression as they entail specific suggestions regarding the etiological development and clinical consequences of MDD cognitive deficits [62]. Moreover, the present review attempted to consider current evidence by analyzing several cognitive dimensions, that are the *cold*, *hot* and *social cognition* domains. Although numerous equivocal and discordant results have been proposed, the present review is the first to have incorporate these concepts within childhood and adolescence. We focused on these developmental stages as we believe that to increase current knowledge on the nature of cognitive disturbances in childhood and adolescence, also considering individuals in at-risk conditions, could represent an actual challenge in a clinical perspective, allowing to early define the clinical phenotypes at enhanced risk of progression to more severe and persistent mental disorders and, most of all, to hypothesize preventive and therapeutic strategies to reduce the impact of these impairments on psychosocial functioning and well-being (Table 1).

In this context, the concept of staging model aims to better characterize the potential manifestations of a mental disorder during a pre-morbid phase [117] with further practice and clinical suggestions. To date, several staging systems have been proposed for various SMI (i.e., for psychoses [118], bipolar disorder [119] and depression [120]), some of which have been already validated for use in clinical practice, with significant therapeutic implications for each stage [121]. Notably, the staging models for psychotic and severe mood disorders identified the *Stage Ia* as a preclinical phase, during which *mild or non-specific symptoms of psychosis or severe mood disorder with mild functional change/decline* [122] could occur, highlights that the manifestations of subtle cognitive disturbances are crucial clinical features during the pre-morbid stage. Thus, cognitive disturbances represent a suitable biomarker that, reflecting causal mechanisms or consequences of the pathophysiology, could be used to consider the possible evolutions of psychopathology during the development of an SMI, suggesting that progression of illness is by no means inevitable, but can be altered by providing appropriate interventions that target individual modifiable risk and protective factors [123]. Moreover, the identification of SMI during the early phases is important for mental health systems’ organization; in fact, the development of *soft entry points* as a milieu integrated in the community and separated from other mental health services has been suggested. These services could be aimed at socialization, educational and vocational support, promoting physical and mental health with the aim to reach the highest number of young people currently at the *Stage Ia* phase, thus referring timely those who start to manifest a more specific subthreshold symptomatology to the appropriate clinical services [121].

However, if extensive evidence has been provided regarding the degree of cognitive impairments across all clinical stages of disorders in the spectrum of schizophrenia, affecting high-risk individuals [15,124], first psychotic episode subjects [125] or long-term course patients [126]), the presence of cognitive deficits in affective disorders, especially in MDD, is less clear. Indeed, although cognitive impairments in BD seem to be less severe than those observed in psychotic disorders [18,127], cognitive disturbances are well documented in BD patients [128], including both first-episode BD [18,129], stable euthymic type I BD [130,131] affecting verbal and visual memory, verbal fluency, executive functioning, attention and processing speed [128,129,130], and also patients living with type II BD [132]. Similarly, much evidence has been found regarding the presence of cognitive impairments prior to the onset of BD [129], including individual at genetic risk [133] or familiar risk [134,135].

On the other hand, regarding MDD, while cognitive impairments have been commonly described during depressive episodes in adulthood [33,34,35,36], current evidence is less clear to define a staging model, especially for the pre-morbid phases encompassing children and the adolescent population, and this is largely due to the limited availability of longitudinal and pre-diagnosis studies, the latter aimed at identifying the presence of cognitive impairments prior to the onset of MDD [60,123]. Indeed, from a developmental perspective, it is not clear if the patterns of cognitive impairments observed in adult individuals with MDD could be extended to youths, since pre-pubertal depression may differ in important ways from post-pubertal depression [42]. In fact, although a lot of evidence has described impairments in different cognitive domains in adolescents with MDD, affecting inhibition capacity, verbal fluency, attention, verbal/spatial memory, working memory, EF, psychomotor and processing speed, negative attentional bias from emotional stimuli, negative interpretation biases and overgeneralized autobiographical memories) [90,136,137,138], other studies failed to clearly detect cognitive impairments in these young populations, suggesting that more research is needed to clarify the relationship between depression and cognitive impairments in children and adolescents [139,140]. Since cognitive impairments in children and adolescents with MDD are very heterogeneous if compared to adult MDD patients, with unclear effect sizes [137], and without a definitive consensus regarding the presence of a specific cognitive profile in subjects at risk for depression [141], an urgent need is to provide an effective early identification of cognitive deficits in childhood and adolescence in the premorbid phase of MDD [137,142]. Thus, it is not surprising that our research was limited in a substantial manner since, excluding the majority of the studies having involved adult populations, only recently the research focus has moved to childhood or adolescence stages, with the idea that these phases are crucial developmental stages associated with heightened risk for the onset of MDD [60]. Considering the *trait*, *scar* and *state* hypotheses, current evidence does not allow to exclusively confirm the validity of one specific theory when childhood or adolescence samples are considered. Similarly, also in the adult population, all the hypotheses regarding neuropsychological profiles in MDD patients received some degrees of support but with equivocal results. If EF impairments were considered a trait-like deficit, processing speed deficits seemed to be explained as a result of a scarring effects, while the overall disturbed neurocognitive profile observed in patients at first MDD episode is considered to be explained in the light of the state-like model [62]. Furthermore, since cognitive impairments were observed in mild, moderate and severe depression, with a direct relationship between depression severity and neurocognitive impairment, some authors were prone to consider that cognitive impairments were more suitably described according to a state-like model [123].

Regarding youth populations and the *trait* hypothesis, as already suggested by Allot et al. [60], equivocal evidence has been derived from premorbid [69,70,71,79] and family studies [63,64,65,66,67] to consider cognitive impairments as a precursor of further depressive episodes. Indeed, while some studies noted that impairments in memory, attention and overall executive functions in youth could represent a trait marker for the development of MDD, other studies failed to confirm the *trait* hypothesis [76,77,78,85,86,91]. At a methodological level, while longitudinal studies without a premorbid neurocognitive assessment have substantially suggested mild trait-like cognitive impairments in youth, discordant results were obtained considering studies with a premorbid cognitive assessment. Indeed, while Singh et al. [67] showed that the impairment of different EF facets could precede the onset of an affective disorder, the studies by Beaujean et al. [86] and Vijayakumar et al. [85] suggested that MDD experienced during adolescence might be associated with cognitive scarring, while Micco et al. [91] recognized EF impairments as linked to a state-line deficits. Thus, a suggestion is to further investigate pre-morbid cognitive functioning with well-designed studies involving longer follow-up periods and by using a more uniform and systematic approach for neuropsychological evaluation. Coherently, a feasible explanation for these equivocal results could involve the heterogeneity in the assessment tools used to detect cognitive deficits in children and adolescent subjects as a trait risk-like factor for MDD development. This is an open challenge in clinical and research settings since also in adult population gold standard measures are lacking to detect cognitive impairments in patients living with MDD [33].

Again, equivocal and discordant results have been found regarding the presence of cognitive disturbances according to a scar-like model in depressed youth or at-risk individuals. Furthermore, as this hypothesis is strictly linked to possible neurobiological dysregulated processes, a main limitation is related to the fact that, to date, no studies in youth subjects are available that involved the use of such biological markers to explain the possible relation between depression and cognitive worsening.

Finally, the presence of state-like impairments in depressed youths also remains unclear. Although studies on the presence of a state-like impairment in depressed youths found deficits in the domains of working memory and processing speed [90], a major limitation is linked to the fact that a state-like impairment cannot be set apart from trait- and scar-based impairments because there is no follow-up assessment when symptoms have resolved [60].

Furthermore, we observed that cognitive assessments in youth and at-risk populations mainly took into account several facets of *cold* cognition, while data regarding possible impairment of *hot* and *social cognition* is substantially lacking. Although *hot* and *cold* cognition have been usually investigated separately within the MDD literature as they were considered as distinct processes, the dynamic interactions between these domains are essential for the maintenance of the depressive cycle (Figure 1) [38]. Indeed, it was suggested that *cold* cognitive impairments, mainly in the executive domain, could act as a gateway, in a trait-like perspective, that could turn into the activation of cognitive biases and maladaptive schemata. Thus, when an acute depressive episode occurs it seems that depressive symptoms could further exacerbate cognitive impairments by turning and incorporating *cold* cognitive deficits into the expression and maintenance of *hot* cognitive biases [38]. Finally, according to the resource allocation theory, it seems that the depletion of cognitive resources allocated to everyday functioning will contribute to broadening these deficits across several *cold* cognitive domains [38,82,110]. Thus, as *hot* and *social cognition* could be considered as other highly valuable markers within the staging model for SMI [123], further studies are needed to elucidate the relationship between MDD and youths at increased risk for its development considering the *hot* cognitive domain impairments. Indeed, as difficulties within social interaction observed in MDD seem to be linked to an altered ability to correctly interpret emotional stimuli and mental states [143], it is not surprising that preliminary evidence suggested that the trait-like vulnerability to depression could be considered as linked to *hot* cognitive impairments in attributing stressful life events to global, stable and internal causes [82]. Moreover, preliminary, albeit contrasting findings, highlighted the possible relation between rumination, as an index of *hot* cognitive impairment, and further deterioration in the *cold* cognitive domain [111,112]. Thus, these findings highlighted the importance to systematically consider the role of cognitive biases as a risk factor for MDD development, posing specific attention to the possible relations between these *hot* cognition deficits to the *cold* cognition impairments [38]. However, although several cognitive vulnerability theories substantially supported the trait-like nature of these *hot* biases [95,106,109,111] to predict depression onset, posing great emphasis on the cognitive consequence of NLEs [81,103,107,108], we found that a systematic assessment of cognitive vulnerabilities that could take in consideration their emergence and consolidation in a neurodevelopmental perspective is still lacking in clinical practice. Thus, further efforts are needed to include the evaluation of *hot* cognitive domain in clinical practice to improve depression screening and detect young individuals in the very early phases of the disorder [109]. To this extent, the definition of a risk classification in a clinical stage perspective should take into account all the cognitive and interpersonal vulnerabilities to depression among youths, helping clinicians to stratify and organize personalized prevention plans to treat depression during adolescence. This preliminary perspective is confirmed by Cohen et al. [109], who demonstrated that promising results could be obtained by improving screening initiatives to identify concurrent depressive episodes, prospective depressive episodes, first lifetime episodes of depression, and recurrent major depressive episodes by incorporating cognitive vulnerabilities assessment (rumination, dysfunctional attitudes, and attributional style) in clinical practice. Indeed, they found that rumination and attributional style emerged as unique and incrementally valid predictors for prospective episodes after controlling for baseline depressive symptoms. Notably, they found that rumination was the only predictor for first lifetime depressive episodes. Moreover, it was found that rumination in early adolescence and attributional style in middle adolescence served as incremental predictors for recurrent major depression [109]. Finally, although evidence is available regarding the involvement of *hot* cognition, to date, no data are available regarding possible alterations of *social cognition* in youth or at-risk subjects, and this represents a major limitation, preventing the clarification of possible relations between this domain and the *cold* cognition in youth.

Though several encouraging findings were observed, the present review has several limitations. Firstly, at a methodological level, it is a qualitative analysis of the current literature. However, we did not consider carrying out a more systematic review as we were initially indented to evaluate whether these issues could be of clinical interest and suitable for further studies with a preventive or therapeutic aim. In fact, unlike other SMIs such as schizophrenia or BD, we observed that studying cognitive impairment in MDD, during the premorbid phase or in childhood/adolescence, is substantially a neglected area of research and only recently the scientific literature is offering new insightful evidence. This observation coincides with the fact that, although useful, staging systems for depression are still not very widespread. Moreover, we believe that it would not be possible to carry out an accurate systematic review since, after a preliminary evaluation, the reviewed studies were characterized by high methodological heterogeneity, particularly in terms of study designs (i.e., fewer longitudinal studies and more studies having involved non-clinical individuals) and of assessment tools used to detect cognitive disturbances among youth. Indeed, a great limitation that negatively influences the possibility of generalizing the studies’ results is to have no such gold standard tools to evaluate cognitive impairments of the *cold*, *hot* and *social cognition* domain. Moreover, this great heterogeneity is also revealed in the theoretical definitions of the cognitive constructs we have analyzed. This probably depends on the mixture of psychological and psychiatric terminologies used that reflects different theoretical backgrounds. For example, while cognitivist psychologists considered the impairments of *hot* cognition labeling them under the term of *cognitive vulnerability*, a medical-oriented approach usually segregated the definition of cognitive impairments according to the definition of *hot* and *cold* cognition as previously used in disorder such as schizophrenia.

In any case, the idea to study the cognitive impairments in a premorbid phase or during childhood/adolescence represents an innovative approach that must be pursued also in MDD with the suggestion that the identification of possible endophenotypes could lead to define specific preventive and therapeutic interventions. However, we know that an individual presenting with subthreshold, cognitive symptoms will not automatically develop a specific full mental disorder as psychopathological trajectories could change over time [144,145,146]. This is the case of individuals at high clinical risk for psychosis, of which it is known that only about one third will evolve over time to a frank schizophrenia disorder [147]. In this context, while other psychiatric conditions or comorbidities should be considered disentangling the psychopathological trajectory, we observed that the evaluation of mental comorbidities as possible confounders is a neglected area in the field of early phases of MDD, especially when researchers are intended to evaluate cognitive functioning of these young subjects [90]. Thus, it is not surprising that Schaefer et al. [78] observed that childhood cognitive functioning did not predict the risk of MDD, unless MDD was accompanied by other comorbid psychiatric conditions. Similarly, Halse et al. [70] found executive functioning impairments as a nonspecific predictor of MDD and other disorders, including anxiety, ADHD, oppositional defiant and conduct disorder in a sample of children and adolescent individuals, while Han et al. [69] found that global EF impairments in adolescent sample could predict an increase of anxious symptoms two years later. Moreover, among these comorbidities, although ADHD would deserve specific interest as it is a common developmental disorder characterized by overall EF impairments [148], only limited evidence is available regarding the role of premorbid executive functioning during childhood as possible predictor of both ADHD and MDD onset [71,73]. Thus, future studies aimed at exploring neurocognition as a trait risk for MDD in youth need to take into account the role of psychiatric comorbidities to avoid possible biases. In addition, another limitation of the reviewed literature is about the lack of evaluation of possible relations between quality of life, daily functioning to cognitive disturbances in depressed youth. These limitations have inevitably negative impacts on the feasibility in offering specific preventive or therapeutic interventions in MDD.

## 5. Conclusions

The present review is the first to have analyzed the role of cognitive impairments during the early phase of MDD in childhood/adolescence and *at-risk* individuals by considering the *trait*, *scar* and *state* hypotheses of MDD by examining the *cold* and *hot* cognitive dimensions. However, we found that this is a substantially neglected area of scientific interest. Indeed, as equivocal and discordant results have been found considering young samples, it is not possible to exclusively confirm the validity of one specific hypothesis. Thus, further studies are needed to better characterize the cognitive dysfunctions in terms of trait, state or scarring manifestations in MDD in childhood and adolescence and in a pre-morbid phase. Several limitations contributed to these inconclusive results, including studies heterogeneity at methodological level, the lack of a systematic assessment of *cold*, *hot* and *social cognition* cognitive domains and their possible interactions in a developmental perspective, the possible contributions of upcoming psychiatric comorbidities and the lack of evaluations regarding the influences of cognitive disturbances on quality of life and daily functioning in youth. Only through a greater understanding of the nature of the cognitive impairments in young populations will become possible to identify clinical endophenotypes at increased risk to MDD and to provide preventive and therapeutic strategies aimed at reducing the impact of these manifestations and improve the quality of life and psychosocial functioning in young people living with MDD.

## Figures and Tables

**Table 1:** Key points to summarize current evidence on the relation between cognition and depression in childhood and adolescence.

Key Points
Cognitive impairments are among the main manifestations of MDD, comprising disturbances in the cold, hot and social cognition domains that negatively influence treatments response, illness course and psychosocial functioning.
Cognitive impairments in MDD could be described according to the *trait, scar and state hypotheses* that considered, respectively, these disturbances as a pre-existing vulnerability marker, a scarring manifestation due to the chronicity/severity of affective episodes, or a temporary feature of an acute depressive episode decreasing with it resolution.
While cognitive impairments have been commonly described in adults with MDD, cognitive impairments in youth still represent a neglected area of scientific interest although this topic is crucial to further identify clinical endophenotypes at increased risked for MDD and to develop preventive and therapeutic interventions in youth.
Unlike schizophrenia or bipolar disorder, only limited and inconclusive data are available considering cognitive impairments in childhood and adolescence during the pre-morbid phase of MDD or even among at-risk individuals, so that current evidence does not allow to confirm the superiority of one specific hypothesis on the nature of cognitive disturbances in youth.
In youth samples, although most of the current data considered cold cognitive domain impairments, disturbances of the hot cognition (i.e., ruminations) might represent another feasible marker to further understand the nature of these deficits during the depressive cycle.
Current limitations regarding cognitive impairments in youth population include heterogeneity of study design, the lack of systematic assessment of *cold*, *hot,* and *social cognition* cognitive domains and of their possible interaction, of their influences on quality of life and daily functioning, and of possible contributions by other psychiatric comorbidities.

## Data Availability

The data presented in this study are available on request from the corresponding author (JL).
